# Strategies to Inhibit Hepatitis B Virus at the Transcript Level

**DOI:** 10.3390/v13071327

**Published:** 2021-07-09

**Authors:** Bingqian Qu, Richard J. P. Brown

**Affiliations:** 1 Division of Veterinary Medicine, Paul Ehrlich Institute, 63225 Langen, Germany; 2 European Virus Bioinformatics Center, 07743 Jena, Germany

**Keywords:** chronic hepatitis B, covalently closed circular DNA, viral integration, transcription factor, nuclear receptor, transcriptional inhibitor, RNA interference

## Abstract

Approximately 240 million people are chronically infected with hepatitis B virus (HBV), despite four decades of effective HBV vaccination. During chronic infection, HBV forms two distinct templates responsible for viral transcription: (1) episomal covalently closed circular (ccc)DNA and (2) host genome-integrated viral templates. Multiple ubiquitous and liver-specific transcription factors are recruited onto these templates and modulate viral gene transcription. This review details the latest developments in antivirals that inhibit HBV gene transcription or destabilize viral transcripts. Notably, nuclear receptor agonists exhibit potent inhibition of viral gene transcription from cccDNA. Small molecule inhibitors repress HBV X protein-mediated transcription from cccDNA, while small interfering RNAs and single-stranded oligonucleotides result in transcript degradation from both cccDNA and integrated templates. These antivirals mediate their effects by reducing viral transcripts abundance, some leading to a loss of surface antigen expression, and they can potentially be added to the arsenal of drugs with demonstrable anti-HBV activity. Thus, these candidates deserve special attention for future repurposing or further development as anti-HBV therapeutics.

## 1. Introduction

In the past decade, global deaths from viral hepatitis have increased to become the seventh leading cause of mortality, annually causing more deaths than AIDS, diabetes, and tuberculosis (1.4 million/year) [1]. Viral hepatitis results in liver inflammation and is caused by hepatotropic viruses, with both acute or chronic disease courses described. These liver-tropic pathogens represent a range of DNA and RNA viruses from diverse viral families with distinct modes of transmission: Hepatitis A virus (HAV), Hepatitis B virus (HBV), Hepatitis C virus (HCV), Hepatitis delta virus (HDV), and Hepatitis E virus (HEV). Both HCV and HBV can cause chronic infections in immune-competent individuals [2], potentially leading to progressive liver injury. According to the World Health Organization, HBV and HCV chronically infect 240 million and 71 million people, respectively. Chronic infections may ultimately result in liver fibrosis, cirrhosis, and hepatocellular carcinoma (HCC). Indeed, an estimated 75% of all HCC cases are attributed to chronic infection with HBV (CHB) or HCV [3,4].

## 2. Basic HBV Molecular Biology

Approximately 90% of HBV virions contain a double-stranded relaxed circular (rc)DNA genome of ≈3.2 kb. Every rcDNA template consists of an intact minus strand covalently bound to a viral polymerase via phosphotyrosine and a partial plus strand [5]. The remaining 10% of HBV virions contain double-stranded linear (dsl)DNA genomes, which are generated from in situ priming of plus-strand synthesis [6]. Upon infection, rcDNA and dslDNA genomes are converted into another form, covalently closed circular (ccc)DNA, by the host’s nuclear DNA repair machinery [7]. The circular genome contains four promoters (core, preS, S, and X), two enhancers (EnhI and EnhII), and encodes four viral genes (precore-core, polymerase, preS-S, and X) [8].

CccDNA serves as a replicative template for all transcripts including precore mRNA, pregenomic (pg)RNA, preS, S, and X mRNA. All transcripts possess a polyadenylated 3′ terminus [9,10]. During replication, these transcripts are translated into seven viral proteins. Precore mRNA is translated and proteolytically processed to yield HBV e antigen (HBeAg), whereas pgRNA is not only reverse transcribed but also translated to form the viral polymerase and HBV core antigen (HBcAg) [11]. PreS and S mRNAs are translated into large-, middle-, and small-envelope proteins. X mRNA is translated to HBx protein [12].

PgRNA is bound to the viral polymerase, and this complex recruits HBcAg dimers for encapsidation [13]. Within nucleocapsids, rcDNA or dslDNA are reverse transcribed into complementary pgRNA. This pgRNA template is then degraded via the RNase H activity of the viral polymerase [14]. Nucleocapsids interact with the envelope proteins, and virions are secreted. Envelope proteins can also be assembled to form non-infectious subviral particles, filaments, and spheres, which do not contain viral DNA genomes [15,16].

## 3. Approved Drugs and Potential Therapeutic Options against Chronic Hepatitis B

Effective treatments for chronic HCV infection are now available and involve combination therapy with direct-acting antivirals (DAAs). DAAs targeting the NS3/4A protease (Voxilaprevir, Paritaprevir, Grazoprevir, Glecaprevir), the NS5A phoshoprotein (Ledipasvir, Velpatasvir, Ombitasvir, Elbasvir, Pibrentasvir), and the NS5B polymerase (Sofosbuvir, Dasabuvir) lead to a sustained virologic response, which is defined as undetectable HCV RNA at 12 weeks post-treatment [17,18,19]. The development of DAAs was initiated and facilitated using two methodologies: high-throughput screening utilizing HCV replicons [20,21] and crystal structures for the viral NS3, NS5A, and NS5B proteins [22,23,24,25,26]. In contrast, due to unresolved viral protein structures and low-throughput in vitro infection-based screening models, no ‘magic bullet’ antiviral treatment that cures CHB has been developed to date.

Currently, CHB is controlled but not cured by approved antivirals. For instance, transcriptionally active HBV DNA in the nucleus is not directly targeted [27]. Except for interferon-α (IFN-α) and pegylated IFN-α, all other licensed drugs are nucleoside (Lamivudine, Clevudine, Entecavir, Telbivudine) and nucleotide analogues (Adefovir dipivoxil, Tenofovir disoproxil fumarate, Tenofovir alafenamide). All these drugs are potent at reducing viral loads and normalizing alanine transaminase levels in CHB patients. However, long-term treatment with many of these drugs leads to the development of multiple drug resistance mutations. In addition, while a limited reduction in cccDNA is achieved, long-term nucleos(t)ide analogue treatment does not reduce hepatitis B surface antigen (HBsAg) levels [28,29,30,31,32].

Both virus and host druggable targets exist at multiple stages of the HBV life cycle, including viral entry, replication, assembly, and the secretion of subviral particles, which do not contain genomic material [33]. For example, myristoylated preS1-derived lipopeptide (Myrcludex B) specifically bound to the human sodium taurocholate cotransporting polypeptide (hNTCP), the bona fide HBV and HDV receptor, prevents HBV entry in urokinase-type plasminogen activator and severe immunodeficient (uPA-SCID) mice repopulated with primary human hepatocytes [34]. Myrcludex B also potently blocked HBV spreading from initially infected hepatocytes to uninfected cells [35]. Although HBsAg levels remained, HBV viral load was significantly decreased at week 24 in the pegylated IFN-α-Myrcludex B cohort (*n* = 7) compared with Myrcludex B monotherapy (*n* = 8) in a phase 1b/IIa trial [36]. For HBV replication, the RNase H domain within viral polymerase has also been effectively targeted by specific inhibitors in previous preclinical trials [37,38,39].

The availability of high-resolution structures for HBV nucleocapsids has facilitated the development of multiple capsid allosteric modulators [40,41,42]. Recently, one leading compound, NVR 3-778, showed reduced HBV DNA and RNA levels in patient serum (*n* = 43) when administered as monotherapy, with a larger reduction observed in combination with pegylated IFN-α (*n* = 10) [43]. Morphothiadin (GLS4), a derivative of heteroaryldihydropyrimidine targeting capsid maturation, showed a potent in vitro antiviral activity and tolerability in healthy participants (*n* = 8) when co-administered with Ritonavir, which boosted plasma concentrations of morphothiadin [44]. More recently, JNJ-56136379 (JNJ-6379) showed good tolerability in treatment-naïve chronic HBV patients in a phase I study. Remarkably, 32% of patients (13/41) had undetectable HBV DNA levels at 4 weeks treatment, despite no alteration in HBsAg levels [45]. In another trial, ABI-H0731 showed safety at 300 mg/day but non-specific side effects at higher doses in some participants. The treatment resulted in dose-dependent declines in both HBV DNA and RNA levels [46].

Two nucleic acid polymers were shown to inhibit the secretion of subviral particles. Both REP 2139 and REP 2165 were well tolerated and showed a substantial activity in treatment-naive patients. The combination of Tenovofir, pegylated IFN-α, and REP promoted HBsAg seroconversion (<0.05 IU/mL) in 60% of patients (24/40). During 48 weeks of follow-up, no viral rebound was observed in 35% of patients (14/40) [47].

All lines of evidence detailed above demonstrate that HBV replication can be controlled, but a permanent cure has not been achieved. Notably, none of the above drugs (except IFN-α) target transcriptionally active templates and decompose viral transcripts. Thus, transcriptionally active templates should be recognized as novel drug targets, and any new-class antivirals targeting this virus life-cycle stage could represent a potential therapeutic option against CHB. Indeed, taking advantage of authentic infection models that allow cccDNA-mediated replication, a number of candidates were identified that either inhibit transcription or impact on the stability of existing viral transcripts. From here onwards, this review chronicles the latest developments and discusses the potential effects of this new class of drugs that could represent components of new therapeutic regimens that have potential as a functional cure for CHB.

## 4. Templates of Transcription: cccDNA and Integrants

HBV chronicity depends on the persistence of two types of viral reservoir, episomal cccDNA and integrants. Upon infection, rcDNA in virions is imported into the nucleus. How rcDNA converts to cccDNA remains unclear, but this appears to solely require the host’s DNA repair machinery [48]. The conversion requires multiple steps: removal of viral polymerase from rcDNA, removal of RNA primer region from the plus strand of protein-free rcDNA, removal of redundant sequences from the minus strand, DNA elongation of both strands, and finally ligation of all DNA ends [49]. Specifically, the host cellular enzyme tyrosyl-DNA-phosphodiesterase 2 (TDP2) likely cleaves the Tyr-rcDNA bond and releases the viral polymerase [50]. Flap structure-specific endonuclease 1 (FEN1) is responsible for the removal of redundant rcDNA-specific structures [51]. Next, DNA polymerase κ and topoisomerases (I and II) catalyze filling in the gap in the plus strand DNA [52,53]. Similarly, polymerase α is involved in the repair of the minus strand during intracellular amplification of cccDNA [54]. Host DNA ligases (LIG1 and 3) mediate the ligation of DNA nicks on both strands, converting it to a closed-circular state, whereas the ligase 4 (LIG4) drives the conversion of dslDNA to cccDNA via non-homologous end joining [55]. Using yeast-extract screening, Wei and Ploss identified five components essential for cccDNA conversion: proliferating cell nuclear protein, replication factor C complex, FEN1, DNA polymerase δ, and LIG1 [56]. These data suggest that cccDNA synthesis depends solely on the host’s machinery and requires the concerted action of multiple nuclear proteins and enzymes, which requires further characterization. Once formed, episomal cccDNA is maintained in the nucleus: cccDNA is long-lived and stable at low copy numbers in non-dividing hepatocytes but sensitive to cell mitosis [57,58].

Distinct from rcDNA, dslDNA in ≈10% of nucleocapsids can be imported to the nucleus to form cccDNA containing a redundant insertion of 16 nucleotides, which can revert to wild-type cccDNA probably via homologous recombination. This was initially observed in duck hepatitis B virus (DHBV) infected ducklings [59]. Alternatively, dslDNA can be integrated into the host genome carrying double-stranded DNA breaks [60]. These integrations occur at rate of one integration per 10^3^–10^4^ hepatocytes in ducklings infected with DHBV and woodchucks chronically infected with woodchuck hepatitis virus (WHV) [61,62] and one integration per 10^4^ hepatocytes/hepatoma cells that were infected in vitro with HBV [63]. Distinct from cccDNA, the HBV integrants are randomly distributed across all chromosomes and show stability during cell mitosis, as integration frequencies in rapidly growing duckling hepatocytes and dividing HepG2^NTCP^ cells post infection were not reduced [61,63,64].

**Figure 1:**
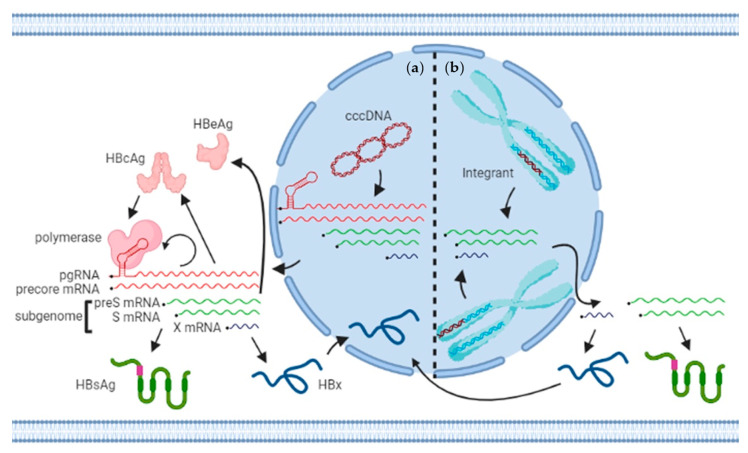
Overview of HBV transcription and translation from cccDNA and genomic integrants. (**a**) In the nucleus where cccDNA resides, RNA polymerase II-mediated transcription from cccDNA generates five capped and polyadenylated transcripts: pgRNA (3.5 kb), precore mRNA (3.5 kb), preS mRNA (2.4 kb), S mRNA (2.1 kb), and X mRNA (0.7 kb), all of which are exported into the cytoplasm. Seven viral proteins are translated from those transcripts (see text for details). (**b**) HBV subgenomic fragments are randomly integrated into different chromosomal locations. PreS/S and probably X transcripts are generated from integrated genomes and translated to HBsAg and HBx. So far, no evidence has emerged that that pgRNA and precore mRNA can also be produced in this manner. HBx is imported in the nucleus and functions as a transactivator on cccDNA.

PgRNA, precore, and subgenomic (preS, S, and X) RNAs are all transcribed from cccDNA (Figure 1a). However, integrated DNA is not capable of producing the 3.5 kilo-base pgRNA and precore mRNA. Nevertheless, both preS and S transcripts are expressed by the integrated DNA (Figure 1b). Indeed, natural HBV integration was identified in the “Alexander” hepatoma cell line, PLC/PRF/5. Four complete and two partial HBV genomes were detected, one of which produces HBsAg, whereas pgRNAs for HBcAg were undetectable [65,66]. HBV integrations were also characterized in other two hepatoma cells, Hep3B (clone F1, 14, and 217) and L6EC3, suggesting that these hepatoma cells with HBV subgenomic integrations are capable of HBsAg production [67,68]. Of note, HBx transcripts are expressed from integration sites in tumor tissues and hepatoma cell lines [69,70]. Lacking a stop codon, the translation of HBx may extend and produce viral–host chimeric genes (e.g., HBx-long interspersed nuclear element (HBx-LINE)), which can promote hepatic damage [71,72].

## 5. Host and Viral Mediators of HBV Transcription

Both stable DNA templates in hepatocytes can be constitutively transcribed. The architecture of the “HBV minichromosome” is composed of one cccDNA template coupled to viral core protein (HBcAg), histones (H3 and H4), and multiple host proteins [73]. This minimal complex was reconstituted in vitro and visualized using electron microscopy [74]. Using chromatin immunoprecipitation, the physical interaction between cccDNA and HBcAg or histones was confirmed [75]. The authors also reported that active cccDNA-mediated transcription parallels the acetylation status of H3 and H4 histones and found that histone deacetylase inhibitors valproic acid and trichostatin A increase cccDNA-bound acetylated histones and HBV transcription. Using deep sequencing of a purified nucleosome fraction, Tropberger et al. demonstrated that high levels of post-translationally modified histones are enriched at specific sites across the entire HBV genome and are associated with efficient transcription, whereas low levels of modified histones are required to bind cccDNA, which represses transcription at the promoters [76]. Regardless of its cccDNA binding capacity, the exact role of HBcAg in transcription from cccDNA remains unclear. HBcAg likely employs its C-terminal domain to maintain transcription and assist in the recruitment of histone acetyltransferases [77].

Efficient transcription requires both ubiquitously expressed and liver-specific transcription factors, as well as additional co-factors [78,79,80]. Host RNA polymerase II initiates cccDNA-mediated transcription. The essential and ubiquitous transcription factors, including IIB, TATA box protein, cAMP response element-binding transcription factor (CREB), CCAAT enhancer-binding protein (C/EBP), and nuclear factor kappa B (NF-κB) not only enhance transcription from cccDNA but also cellular genes essential in cell differentiation, proliferation, and survival. Inhibitors targeting these would be not specific against HBV transcription and would likely induce unwanted effects on cells.

One of the largest families of liver-specific transcription factors, belonging to the nuclear receptor superfamily, is the hepatocyte nuclear factor (HNF). HNF1α and other HNF1 members form homo/heterodimers, which increase the transcriptional activity of preS, X promoters, and enhancer II activity [81,82,83]. When it activates the NF-κB pathway, HNF1α in turn inhibits transcription [84]. HNF3α/β/γ homo/heterodimers bind the preS promoter, enhancer I, enhancer II, and upregulate their transcriptional activity [85,86,87]. HNF4α targets mainly the core promoter and enhances its transcription in hepatoma cells but not HeLa cells [88]. In contrast, liver-enriched HNF6 inhibits transcription of the S promoter but mediates no effects on other promoters or enhancers [89]. Currently, agonists and antagonists targeting any of the above HNFs are unavailable. It was discussed whether HNF4α could represent a potential drug target as it contains a ligand-binding pocket [90]. How to position a drug specifically onto HNF proteins interacting with HBV cccDNA will be a major challenge in the future development of this potential class of inhibitors.

**Figure 2:**
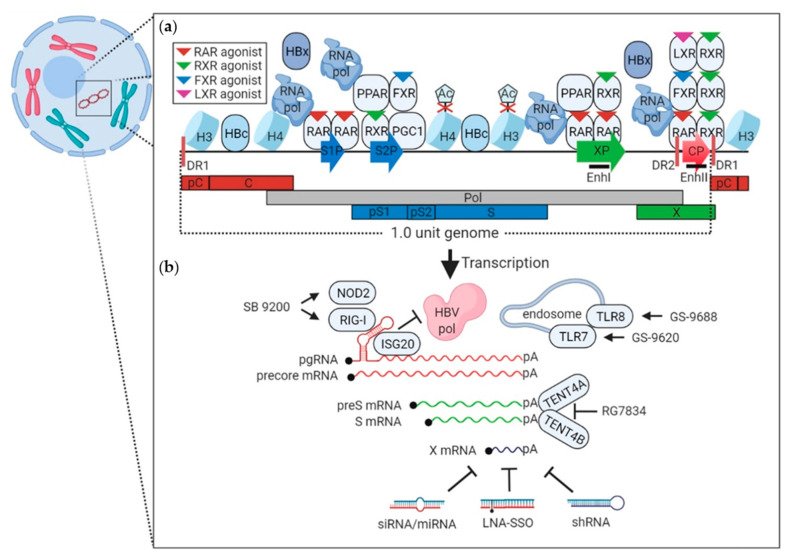
Approaches to inhibit transcription and interfere with viral transcripts. (**a**) The 1.0 unit genome is packaged with essential host factors, histone 3 (H3) and 4 (H4), RNA polymerase (RNA pol), as well as associated HBcAg (HBc) and HBx. Agents that block the acetylation of H3 and H4 (red cross) repress general transcription. Homodimers (i.e., RAR-RAR) and heterodimers (i.e., RAR-RXR, FXR-RXR, LXR-RXR, etc.) bind specific promoter and enhancer regions and maintain transcription. When RAR, RXR, FXR, and LXR agonists (shown as inverted triangles and summarized in Table 1) bind these nuclear receptors, transcription from the HBV promoter is suppressed. Core (CP), preS (S1P), S (S2P), and X promoters (XP) are shown as arrows overlaid onto the 1.0 unit HBV genome. Each promoter controls the transcription of core (pC-C, shown in red), polymerase (Pol, in gray), S (pS1-pS2-S, in blue), and X (in green) open reading frames, respectively. Two enhancers (EnhI and EnhII, thick black line) are marked along with direct repeat elements (DR1 and DR2, thick red line). (**b**) TLR7 (i.e., GS-9620) and TLR8 agonists (i.e., GS-9688) induce endosome-mediated sensing of single-stranded viral transcripts. Targeting pgRNA (red), ISG20 prevents HBV polymerase (pol) binding to the pgRNA, while RIG-I/NOD2 agonists (i.e., SB 9200) promote RIG-I specific binding to the ε region in the pgRNA, which competes for polymerase-pgRNA association and subsequent virion encapsidation. PreS and S mRNAs (green) are stabilized by host enzymes TENT4A/B (PAPD5/7), which can be inactivated by a specific inhibitor, RG7834. In addition to small molecules, RNA interference mediates the degradation of all HBV transcripts including X mRNA. Locked nucleic acids (LNA-SSO), small hairpin RNA (shRNA), small interfering RNA (siRNA), as well as microRNA (miRNA) have been developed to bind viral transcripts, mediating their decay.

With forty-eight members, nuclear receptors (NRs) are the most abundant superfamily of transcriptional mediators in metazoans. They are mainly triggered by ligand–receptor interaction and function as transcription factors involved in cell proliferation, metabolism, and homeostasis [91]. NRs share a canonical structural organization with one or more variable transactivation domains. These include a conserved DNA-binding domain, a flexible hinge-containing nuclear localization signal, and a ligand-binding domain at the C-terminus. When they are stimulated by endogenous or exogenous ligands, the receptors dimerize either as homodimers or heterodimers (two proteins among the same subfamily, e.g., RARα–RARβ). Frequently, they also form heterodimers with two components from different subfamilies (e.g., LXRβ-RXRα, FXR-RXR, PPARγ-RXR, etc.), as shown in Figure 2a [92,93,94]. In terms of known binding profiles between the receptors and ligands, small molecules imitating the ligands can be simply generated by computer-aided drug design. Indeed, this feature makes NRs attractive as potential drug targets. The strategies for targeting NRs are described in detail in the following section.

HBV encodes HBx as a viral transactivator and uses this to promote transcription from cccDNA. Expressed early post infection, HBx does not directly bind cccDNA but is recruited onto the minichromosome and modulates the stability of acetylated histones. In the case of HBx-minus HBV mutants, histones are hypoacetylated, while the recruitment of histone deacetylases histone deacetylase 1 (HDAC1) and sirtuin-1 (SIRT1) are profoundly increased [73]. Furthermore, mapping analysis determined that C-terminally mutated HBx recruits less acetyltransferase p300 and more HDAC1 onto cccDNA templates [95]. HBx not only initiates but also maintains transcription from cccDNA. Lentiviral trans-complementation of HBx rescued active transcription from the HBx-minus virus for weeks post infection, confirming that HBx but not other viral proteins promote viral transcription [96]. In parallel, HBx physically binds DNA damage-binding protein 1 (DDB1) [97]. This complex further hijacks Cullin 4, which is a component of host E3 ubiquitin ligases, and mediates ubiquitin–proteasomal degradation of host restriction factor structural maintenance of chromosome 5/6 (SMC5/6) on the cccDNA [98,99]. However, how SMC5/6 blocks transcription from cccDNA remains unclear. SMC5/6 may recognize an HBV-specific sequence motif, but this seems unlikely, as HBx-mediated augmentation of gene transcription is limited to extrachromosomal DNA templates: this phenomenon is not observed for HBV genomes with identical sequences integrated into host chromosomes [100]. Another possibility is that SMC5/6 senses the supercoiled cccDNA at transcription initiation and topologically fixes cccDNA in the supercoiled state, at which point transcription elongation is not possible. Although SMC5/6 shapes the topology of normal chromosomes, it plays no obvious role in the transcription of cellular genes.

Current knowledge of gene transcription from HBV genomic integrants remains limited. HBV integrants are randomly distributed throughout the entire host genome, without any evidence for preferential integration sites or adjacency to specific gene classes. The transcription from integrants is independent and not associated with cccDNA-mediated viral replication, since there is no difference in HBeAg positive and negative patients [101,102]. Indeed, preS/S transcripts generated from integrants but not episomes become dominant in HBeAg-negative chimpanzees [103]. Similar to episomal cccDNA, the transcription process also is driven by preS/S promoters and regulated by HBV enhancers. However, depending on how close the integration site is to cellular enhancers or repressors, transcription efficiency may differ between individual integrants. Some integrants transcribe a C-terminally truncated HBx viral-cell fusion gene (i.e., HBx-LINE). Despite the lack of its C-terminus, the HBx in the fusion still retains transactivation activity [104,105], because deletion of the C-terminal 14 amino acids (residues 141–154) does not abolish that function [106]. Furthermore, random insertion of the foreign HBV genome into host genomes may result in genomic instability and transcriptional perturbations affecting multiple genes [107,108]. The function of affected genes can determine progression to carcinogenesis [109].

**Table 1:** Inhibitors targeting HBV transcription.

Substance	Property	Target	Clinical Status	IC_50_ HBV Transcription	Inventor	Reference
IFN-α	Cytokine	STAT1/2,histones	Approved	180 μg/week ^1^500 IU/mL	Merck ^2^	[110,111]
rIL-6	Cytokine	STAT3,HNF1α/4α	Preclinical	20 ng/mL	n/a	[112,113]
TGF-β	Cytokine	HNF4α,AID	Preclinical	10 ng/mL	n/a	[114,115,116]
Tazarotene(Tazorac)	Small molecule	RARβ/γ	Approved ^3^	20–75 nM	Allergan	[117]
Tamibarotene(Am80)	Small molecule	RARα	Approved ^3^	≈1 nM	Nippon Shinyaku	[118]
Isotretinoin(Accutane)	Small molecule	RAR/RXR	Approved ^3^	1.2 μM	Roche	[119]
Bexarotene(Targretin)	Small molecule	RXRα	Approved ^3^	1–5 μM	Ligand Pharma	[120]
GW4064	Small molecule	FXRα	Preclinical	0.2 μM	GSK	[121,122]
EYP001	Small molecule	FXR	Phase II	1.25–2.5 μM	Enyo Pharma	[123]
T0901317	Small molecule	LXRα/β	Preclinical	0.3–3 μM	Merck	[124]
GW3965	Small molecule	LXRα/β	Preclinical	0.3–3 μM	GSK	[124]
MLN4924	Small molecule	NAE1	Phase II/III ^3^	290 nM	Takeda	[125,126,127]
Nitazoxanide	Small molecule	HBx-DDB1	Approved ^3^	20 μM	Romark Lab.	[128,129]

^1^ Dose for patients according to the AASLD guideline in 2016. ^2^ Referring to in particular Intron A (IFN-α-2b). ^3^ Approved for administrations other than chronic HBV infection. IC_50_: half maximal inhibitory concentration in in vitro models; IFN: interferon; IL: interleukin; STAT: signal transducer and activator of transcription; TGF: tumor growth factor; HNF: hepatic nuclear factor; AID: activation-induced cytidine deaminase; RAR: retinoic acid receptor; RXR: retinoid X receptor; FXR: farnesoid X receptor; LXR: liver X receptor; NAE1: NEDD8-activating enzyme 1; DDB1: DNA-damage binding protein 1. GSK: GlaxoSmithKline. n/a: not applicable.

## 6. Strategies Targeting Viral Transcription

### 6.1. Cytokines

An approved antiviral therapeutic, IFN-α does not directly inhibit viral replication but stimulates the induction of interferon-stimulated genes (ISGs), the protein products of which possess broad antiviral activity. Targeting multiple steps in the HBV life cycle, ISGs exert a range of distinct mechanisms to control HBV replication [130]. IFN-α treatment induces STAT1/2 recruitment onto cccDNA that represses transcription. In addition, the cytokine inhibits transcription from cccDNA by modulating its epigenetic modification both in vitro and in humanized mice. IFN-α leads to histone hypoacetylation at the H3K9 and H3K27 residues and recruitment of transcriptional repressors such as HDAC1 onto the cccDNA (Table 1) [110,111]. One of the ISGs, apolipoprotein B mRNA editing enzyme catalytic subunit 3A (APOBEC3A), binds HBcAg to facilitate attachment to cccDNA [131]. Deaminated cccDNA via APOBEC3A is prone to degradation by the hydrolysis of nuclease ISG20 (Stadler et al., in press). Moreover, IFN-induced tripartite motif 22 protein (TRIM22) inhibits core promoter activity and viral gene expression both in vitro and in vivo [132].

Pro-inflammatory interleukin-6 (IL-6) inhibits pgRNA and preS/S transcription from cccDNA in infected HepG2^NTCP^ cells. Recombinant IL-6 (rIL-6) treatment dissociates HNF1α and HNF4α with cccDNA, both of which are required for transcription as described above. rIL-6 induces the overall phosphorylation of STAT3; however, lower levels of phospho-STAT3 are recruited onto the cccDNA compared to IL-6 cellular target genes. This redistribution results in decreased HBV transcription [112].

Transforming growth factor (TGF-β) treatment induces the expression of activation-induced cytidine deaminase (AID), and to a lesser extent, APOBEC3F and 3G but not APOBEC3A. AID associates with HBV RNA and can be incorporated into nucleocapsids, where viral pgRNA gets deaminated [114]. AID-mediated HBV reduction takes place in a polymerase-dependent manner, which requires a physical association between AID and the polymerase. This interaction further mediates a recruitment of RNA exosome proteins [115]. Similar to IFN-α and IL-6, TGF-β also significantly downregulates HNF4α expression and core promoter activity, which leads to a reduction in the amount of pgRNA [116]. These findings identify HNF (especial HNF4α) as an attractive drug target. Agents blocking HNF activity or dissociating it from cccDNA would potentially inhibit viral gene transcription from cccDNA.

### 6.2. Retinoic Acid Receptor Agonists

Efficient transcription from cccDNA relies heavily on NRs that bind to response elements in the promoters or enhancer regions in a sequence-dependent manner [133]. For instance, the retinoic acid receptor (RAR) homodimers and heterodimers recognize the DNA element 5′-(A/G)G(G/T)TCA-3′ located in the core, preS, and X promoters (Figure 2a). If a ligand binds the receptor, transcription from these promoters is repressed. Likewise, small-molecule RAR agonists also exert transcriptional inhibition.

Tretinoin (all-trans retinoic acid), Acitretin, Adapalene and Tazarotene were identified by screening a library of FDA approved drugs. The most potent hit, Tazarotene, inhibits transcription from cccDNA with IC_50_ values of 20–75 nM without reducing cccDNA levels. It targets mostly RARβ but not RARα and RARγ in infected primary hepatocytes [117]. Nkongolo et al. identified Tamibarotene (Am80) using another FDA-approved drug library and further compared Tamibarotene (RARα-specific) with other agonists: Tazarotene (RARβ -specific), Adapalene (RARγ-specific), and all-trans retinoic acid (pan-activity). Remarkably, Tamibarotene inhibits transcription selectively from cccDNA in primary hepatocytes but shows no effect on the transcription from integrants in HepG2.2.15 cells [118]. These studies suggested that RAR agonists targeting one subunit in the dimer would be sufficient to shut down gene transcription from cccDNA. Of note, RAR also modulates NTCP promoter activity. RARα stimulation by Ro41-5253 prevents NTCP expression and diminishes hepatocyte permissiveness to HBV infection [134].

RAR agonists have shown promising in vitro activity. However, Birkus et al. compared twenty RAR agonists in vitro and selected Accutane for in vivo experiments using PXB humanized mice that were treated twice per day with 30 mg/kg Accutane for 28 days. Neither HBV DNA nor HBsAg levels were reduced by Accutane in vivo [119]. In a parallel study, we treated uPA-SCID humanized mice with 1 mg/kg Tamibarotene daily for 14 days (in vitro IC_50_ value: 1 nM). In agreement with Birkus et al., HBV DNA and HBsAg levels were not altered (data not shown). It is unclear whether these agonists administered at tolerable doses induce effective RAR responses in the liver.

### 6.3. Retinoid X Receptor Agonists

Associated with peroxisome proliferator-activated receptor (PPAR) or farnesoid X receptor (FXR), retinoid X receptor (RXRα) heterodimers bind enhancers I and II at putative DNA elements 5′-TGAACCTTTACCC-3′ and 5′-CTGAACCTTTACCC-3′, respectively [135,136]. RXR-specific agonist Bexarotene inhibits the transcription of pgRNA and other viral RNAs in HepG2^NTCP^ (IC_50_ value: 1–5 μM), HepaRG (≈5 μM), and primary tree shrew hepatocytes (≈5 μM). Moreover, transient or stable silencing of RXRα expression enhances HBV replication. RXRα disruption is accompanied by the genetic downregulation of arachidonic acid and eicosanoid pathways, which is responsible for hepatic lipid metabolism [120]. The contribution of these metabolic pathways to the efficacy of Bexarotene’s antiviral effect requires further characterization.

### 6.4. Farnesoid X Receptor Agonists

FXRα is a nuclear receptor activated by its natural ligand, bile acids [137,138]. It interacts with PPAR or RXRα to form heterodimers. The dimer binds to a particular DNA response element 5′-AGGTCANTGACCT-3′ located at enhancer II and S promoter and thereby transactivates several genes involved in hepatic lipid and bile acid metabolism [139,140]. It is worth noting that bile acids activate the FXR-RXR heterodimer that transcribes Src homology region 2 domain-containing phosphatase-1 (SHP-1). SHP-1 can inactivate the RAR-RXR heterodimer and prevent the expression of NTCP. Due to negative feedback, down-regulated NTCP in turn lowers the influx transport of bile acids [141]. Therefore, both FXR-RXR and RAR-RXR receptors might play essential roles in bile acid metabolism and HBV replication.

FXR agonist GW4064 and a bile salt derivative 6ECDCA inhibit the expression of viral mRNA, with an IC_50_ value of 0.2 μM, and reduce cccDNA levels in differentiated HepaRG cells. GW4064 treatment reverses most of the HBV-upregulated FXR gene expression [121]. Further investigation has demonstrated that GW4064 treatment affects cccDNA levels for both wild-type and HBx-minus viruses, which was consistent with the concept that HBx is not involved in cccDNA formation and maintenance. However, GW4064-induced inhibition of pgRNA and precore mRNA transcription is HBx dependent [122]. These data imply that HBx likely interacts with FXR heterodimers in the minichromosome. Another agonist, EYP001, has been validated in preclinical and phase I trials. The drug was well tolerated in healthy volunteers, reduced HBV DNA and HBsAg levels in HepaRG cells, and showed an additional effect on HBV DNA in combination with Entecavir [123].

### 6.5. Liver X Receptor Agonists

Liver X receptor (LXR) is a pivotal regulator in lipid and cholesterol metabolism, with at least two subtypes, LXRα and LXRβ, both of which interact with RXR. The heterodimer binds specific DNA element 5′-AGGTCANNNNAGGTCA-3′ and regulates the transcription of multiple genes [142]. Recently, LXR agonists have been tested for anti-HBV activity. Two agonists T0901317 and GW3965 but not antagonist SR9238 potently inhibited the gene expression and transcription of viral RNAs in HepaRG and primary hepatocytes, although cccDNA levels were not substantially reduced. Remarkably, no significant antiviral activity was observed in HepG2^NTCP^ cells [124]. These data suggest that the metabolic profile in HepG2 hepatoma cells might be distinctly different from that in primary hepatocytes. Both agonist treatments decrease cholesterol 7α hydroxylase 1 (CYP7A1) mRNA levels. Silencing CYP7A1 inhibits HBV replication in primary hepatocytes, illustrating that CYP7A1 is a host dependency factor associated with the LXR pathway.

### 6.6. Inhibitors Acting on the HBx-DDB1 Complex

Two independent studies have demonstrated that HBx, as the sole HBV-encoded transactivator, can be targeted by small molecules. Qu et al. reported that MLN4924 (Pevonedistat) inhibits HBV replication with IC_50_ values as low as 62 nM and further characterized it as a transcriptional inhibitor of genotype D HBV-infected HepG2^NTCP^, HepaRG^NTCP^ cells, and primary hepatocytes. Notably, MLN4924 selectively reduces the enhanced transcription by lentiviral HBx expression at the baseline level of that of HBx-minus virus, suggesting a dependency on HBx [125,126]. Meanwhile, Sekiba et al. found that MLN4924 restores SMC5/6 protein expression and reduces viral transcription in the HBV minicircle system and in primary hepatocytes infected with genotype C HBV [127]. These side-by-side studies identify MLN4924 as a promising transcriptional inhibitor.

Nitazoxanide, as well as its metabolite tizoxanide, has a broad antiviral activity toward HBV and HCV [143]. Using a split luciferase system that allows a screening of agents interfering with HBx-DDB1 association, Sekiba et al. identified Nitazoxanide in an FDA-approved drug library. The drug inhibits viral RNA transcription in HepG2 cells transfected with HBV minicircles and HepAD38 cells with integrants. However, Nitazoxanide suppresses viral transcription in primary hepatocytes infected with genotype C HBV only at a very high dose (20 μM) [128]. Such an in vitro dose will limit further clinical development. Furthermore, Nitazoxanide at high doses was unable to completely block HBx-DDB1 binding, although the binding was reduced as shown by a co-immunoprecipitation assay. These disadvantages indicate that Nitazoxanide is not a viable candidate for further clinical development.

## 7. Strategies to Degrade Existing Transcripts

### 7.1. Interferon Stimulated Genes (ISGs)

In addition to its function in cccDNA degradation, ribonuclease ISG20 directly binds to the ε region of viral pgRNA and degrades it in the presence of co-factors (Figure 2b) [144,145]. Further study characterized that the N6 methyladenosine modified viral transcripts are selectively sensed by ISG20 and processed for degradation [146]. IFNα-induced zinc finger proteins trigger viral RNA decay in vitro and in a transgenic mouse model [147,148]. One adaptor protein in the Toll-like receptor pathway, myeloid differentiation primary response protein 88 (MyD88), facilitates the decay process [149]. IFNα inducible myxoma resistance protein 1 (MxA) impedes pgRNA encapsidation by interacting with HBcAg [150,151].

### 7.2. Terminal Nucleotidyltransferase

Terminal nucleotidyltransferase 4A (TENT4A) and 4B (TENT4B) proteins (also termed PAPD7 and PAPD5) are non-canonical poly(A) RNA polymerases that generate “mixed tails” of various nucleotides at the 3′ termini of RNAs by means of non-templated addition to protect mRNA from deadenylation. HBV hijacks this machinery via exploiting the TENT-ZCCHC14 complex and creates mixed tailing for protection as characterized by TAIL-seq [152]. HBV gene expression is severely impaired, when TENT4A and TENT4B are simultaneously knocked down. In particular, knockdown of the TENTs results in the destabilization and degradation of HBV mRNAs without affecting the production of viral pgRNA transcripts [153]. RG7834, belonging to the dihydroquinolozinone chemical family, was identified to target catalytic domains of both TENTs. RG7834 has an IC_50_ value of 2–6 nM in vitro and shows a selective reduction in HBV mRNAs and HBsAg. In addition, RG7834 given orally to humanized mice leads to a mean 1.09 log-scale HBsAg reduction compared to Entecavir treatment (Table 2) [154]. In line with this finding, Hyrina et al. identified that ZCCHC14 together with TENTs stabilize HBsAg expression via 3′ RNA tailing, but so far, no inhibitor targeting ZCCHC14 is available [155].

### 7.3. Innate Immune Agonists

As one of the pathogen-associated molecular patterns, foreign RNA can be recognized by innate immune sensors, including TLR7/8 (specific for ssRNA), TLR3 (for dsRNA), RIG-I (for 5′ triphosphate RNA), and MDA5 (for long dsRNA) and so on [178]. TLR7 agonist GS-9620 has been extensively studied. GS-9620 shows marked and sustained reduction in viral load in woodchucks chronically infected with WHV and chimpanzees infected with HBV [179,180]. Surprisingly, no significant clinical changes in viral DNA and HBsAg levels were observed in CHB patients in phase Ib/IIa trials [181,182]. Without a direct antiviral activity, GS-9620 can induce IFN-α, other cytokines, as well as intrahepatic T-cell and B-cell aggregates [157,158]. Although they enhance HBV antigen presentation, overall immune responses are likely not sufficient to counteract HBV replication in patients. Treatment with media from PBMCs stimulated with TLR8 agonist GS-9688 shows a reduction in viral markers in primary hepatocytes. In woodchucks, treatment reduces intrahepatic WHV DNA and RNA levels by 20-fold [183,184]. A pan-TLR7/8 agonist, RG7854, is also under evaluation (Table 2).

RIG-I has a dual action against HBV replication. RIG-I senses the 5′ ε region of viral pgRNA and induces type III interferon production. Through binding the ε region, RIG-I also competes with the interaction between ε and viral polymerase, which substantially suppresses polymerase-mediated encapsidation (Figure 2b) [185]. Based on these findings, RIG-I/NOD2 agonist SB 9200 (Iranigivir) was developed and evaluated. SB 9200 treatment lowers serum and hepatic levels of WHV DNA and intrahepatic levels of viral RNA in woodchucks [163].

A major limitation of innate immune agonists is that PBMCs rather than infected hepatocytes exhibited better responses. The agonists elicit IFN-α and ISG production, which eventually modulates hepatocytes via an immune-regulatory mechanism. However, the hepatocytes frequently exposed to a quantity of foreign RNA from digested foods and intestinal microbiota have established a strategy that limits the expression levels of the sensors to avoid excessive innate sensing. Furthermore, the cytokines secreted by PBMCs may lead to unexpected and potentially damaging immune responses in the liver.

### 7.4. shRNA and siRNA

Oligonucleotide-based inhibitors are maturing as therapeutics. The majority of these are either small RNAs mediating their antiviral effect via the RNA-induced silencing complex (RISC) or single-stranded DNA-like molecules recruiting RNase H-mediated mRNA degradation. In one of the first studies examining their efficacy, Huh7 cells or mice were co-transfected or hydrodynamically injected, respectively, with one HBV-expressing plasmid and a second plasmid encoding small hairpin RNA (shRNA) homologous to viral mRNAs [165]. Next, HepG2.2.15, HepAD38, and HepAD79 cell lines carrying stable HBV integrants were transfected with synthesized small interfering RNA (siRNA) [186,187,188]. One of the candidates, HBVU6no.2, which specifically binds to the S region, led to a reduction of pgRNA and preS/S mRNAs, as confirmed by Northern blotting. Secreted HBsAg was reduced by 94% in transfected cells and 84% in mouse serum upon RNAi treatment, while intrahepatic HBcAg was also reduced by >99% [165]. Furthermore, another candidate, shHBV765, was evaluated using HBV transgenic mice transduced with recombinant adenovirus encoding these targeting shRNAs. The treatment diminished preS/S transcripts and, to lesser extent, pgRNA, on day 26 in mice [167]. These early studies suggested that active transcription, not only from an episomal plasmid but also from viral genomic integrants, could be successfully targeted upon shRNA and siRNA treatments.

A major problem to extend the use of first-generation small RNAs as a therapeutic is the scarcity of effective liver-specific delivery. To solve it, preferential RNA delivery into hepatocytes requires carriers (liposome, nanoparticles, etc.) and conjugates to enter the hepatocytes, to adapt to the cellular environment and to avoid endosomal sequestration [189]. Cholesterol-conjugated siRNAs (chol-siRNA) enable transmembrane delivery into the cytosol via membrane fusion. Chol-siRNA is further modified with a disulfide bond, a carboxy dimethylmaleic anhydride (CDM), and a tri-antennary N-acetylgalactosamine cluster (GalNAc). GalNAc mediates hepatocyte targeting via its binding to the hepatocyte-specific expressed asialoglycoprotein receptor (ASGPR). After ASGPR-mediated endocytosis, the unstable acidic CDM is hydrolyzed in the endosome, destabilizing the endosome. When the disulfide bond is cleaved, the small RNA escapes from the endosome and is released in the cytosol.

Modified chol-siRNAs exhibit more than 500-fold improvement in knockdown efficacy compared to the chol-siRNA alone. Accordingly, Wooddell et al. designed ARC-520, which is composed of two different chol-siRNAs (chol-siHBV-74 and -77) and one CDM-GalNAc labeled peptide [169,170]. In these experiments, ARC-520 showed safe and efficient activity in reducing viral RNA levels in mice injected with HBV plasmids, HBV transgenic mice, and cynomolgus monkeys [170]. However, upon ARC-520 treatment, while HBsAg levels were profoundly reduced in treatment-naive HBeAg positive patients, smaller reductions were observed in patients who had received nucleos(t)ide therapy or were HBeAg negative. Experiments in chimpanzees further suggested that this reduced efficacy is due to transcriptionally active integrants lacking 3′-end target sites for two siRNAs in ARC-520 [103,190]. Using similar strategy, GalNAc-modified siRNA candidates (summarized in Table 2: JNJ-3989, VIR-2218, RG6346, etc.) were designed and evaluated in clinical trials. Data released after the ongoing trials will broaden our understanding of the efficacy and potency of these inhibitors.

### 7.5. Single-Stranded Oligonucleotides

An alternative strategy is to employ single-stranded DNA-like oligonucleotides (SSO), which are typically 12 to 20 mers in length. They bind the mRNA target and mediate its degradation by via RNase H activity. These oligonucleotides are often modified with locked nucleic acids (LNA), which are nucleotide analogs that contain a methylene bridge between the 4′-carbon and the oxygen atom at 2′-carbon in the ribose ring. This stabilizes the whole structure and improves intracellular stability [191]. In an early study, LNA-modified oligonucleotides were labeled with Alexa546 fluorescein and designed to target pgRNA at the 5′ terminus. By lipofectamine transfection, the LNA-Alexa oligonucleotides showed exclusive nuclear localization and potent inhibition of viral pgRNA at 24 h post transfection in HepG2.2.15 cells [175]. Furthermore, one antisense oligonucleotide was selected to target the most conserved region among all the HBV genotypes in the X open reading frame. Unmodified oligonucleotides are unstable [192]; however, modified ones are safe, stable, and potent. They have shown dose-dependent inhibitory effects on viral replication from integrants in HepG2.2.15 cells and transgenic mice. Moreover, the treatment also leads to a 10-fold reduction in transcription from episomal templates, including cccDNA in infected Huh7^NTCP^ cells and 1.3 genomic unit plasmids injected into immunodeficient mice [193]. Recently, Javanbakht et al. engineered LNA-SSO and further conjugated it to three GalNAc moieties to facilitate specific binding to the liver-specific ASGPR. Strikingly, the GalNAc-LNA-SSO has shown remarkably high potency in AAV-HBV transduced mice and resulted in a sustained >1000-fold reduction in HBsAg expression [168]. So far, there are two more oligonucleotides available in phase II trial as summarized in Table 2. Since most oligonucleotide-based inhibitor trials have undisclosed data, it is not currently possible to compare their efficiencies.

### 7.6. MicroRNAs

Finally, mature microRNAs (miR) processed from a pre-miR precursor can recruit Ago and Dicer proteins, assemble the RISC complex, and drive the degradation of the mRNA target containing an miR-complementary sequence. It is also possible to transfect an exogenous miR mimic that functions similarly to the authentic miR. MiR-122 is a liver-enriched miR that plays essential roles in the maintenance of HCV RNA replication. MiR-122 binds seed sites present in the 5′-untranslated region and stabilizes HCV genomes [194]. Therapeutic silencing of miR-122 in primates leads to long-lasting suppression of HCV replication [195]. However, whether miR-122 interacts directly with HBV transcripts remains unclear. It has been reported that miR-122 can inhibit viral pgRNA expression, whereas silencing it leads to increased HBV production. MiR-122 induces downregulation of heme oxygenase-1 (HO-1), and knocking HO-1 down results in an increase in HBV replication. However, whether HO-1 mRNA is an authentic target of miR-122 was unclear [176]. Another study demonstrated that HBx interferes with p53 binding to core promoter and enhancer I via miR-122 downregulation; hence, this increases HBV transcription [177]. Unveiling cellular target(s) of miR-122 in infection models will help us to evaluate whether therapeutic use of a miR-122 inhibitor affects HBV gene transcription.

## 8. Unanswered Questions

### 8.1. What Is the Next Step for the Development of Nuclear Receptor Agonists?

Given that many NR agonists have been approved for diseases other than CHB, their safety in humans is already established. However, how to enhance their efficacy and specifically target them toward hepatocytes requires further optimization. As summarized in this review, RARα/β/γ and FXRα agonists have the lowest in vitro IC_50_ values (<100 nM), which suggests that both RAR and FXR heterodimers play essential roles in HBV infection and bile acid metabolism (Table 1) [141]. Despite a potent in vitro effect, in vivo experiments have shown that Accutane was unable to reduce HBV DNA and HBsAg levels in PXB mice repopulated with human hepatocytes and treated daily with 60 mg/kg for 28 days. Surprisingly, RNA-seq analysis revealed that Accutane treatment leads to different transcriptome profiles in fresh primary human hepatocytes versus those that were transplanted into PXB mice after 28 days [119]. These data suggest that mouse liver environment likely reshapes the metabolic profile of human hepatocytes and makes them insensitive to RAR agonists. Furthermore, these data indicate that liver metabolic systems may exhibit species specificity, and therefore, humanized mice might not represent the most suitable model for the evaluation of NR agonists. In line with this, Brown et al. recently developed a genetic modified mouse model that provides a more human-like metabolic profile in the murine liver, facilitating improved replication of HCV. The ablation of mCd302 in humanized murine hepatocytes modulates the transcriptional landscape, influencing the expression of multiple components involved in NR signaling [196]. Therefore, this model system may also have potential for development as an HBV drug-screening platform.

### 8.2. Does Preventing HBx-Dependent Transcription from cccDNA Lead to a Complete Repression?

So far, neither MLN4924 nor Nitazoxanide was extensively evaluated in vivo. The sole pilot clinical trial of Nitazoxanide showed that viral loads became undetectable in eight of nine participants and HBsAg seroconversion occurred in three of nine participants as early as 8 weeks upon treatment [129]. Future trials should focus on a larger patient cohort and include all necessary control arms. To achieve a complete repression, blocking HBsAg production from viral transcripts arising from both cccDNA and integrants is required; however, the latter is not controlled by HBx protein. Transcription from viral genome integrations is not sensitive to MLN4924 treatment (Qu and Nebioglu et al., manuscript in submission). Of note, the transcriptional activity of HBx-minus virus is 50–100 fold reduced compared to wild-type virus. If HBx-mediated cccDNA transcription is completely suppressed by MLN4924 and Nitazoxanide, the virus would stay in a “low transcriptional status”. Both treatments target the HBx-host factor complex but do not directly degrade HBx [96]. Although targeting HBx cannot lead to a complete repression, candidates that show potency directly against HBx could be clinically viable and act as “add-on” treatments in combination to nucleos(t)ide analogs and other therapeutics.

### 8.3. Are Differences between mRNA from cccDNA, mRNA from Integrants, and Cellular mRNA Transcripts Relevant?

The selective effect of TENT4A/B upon RG7834 treatment may be dependent on the differences between cellular and HBV RNA at their 3′-termini. Cellular mRNAs often contain two polyadenylation sequences (AAUAAA and AUUAAA) and generate canonical poly(A) tails [197], whereas HBV poly(A) signals contain the sequence UAUAAA, which promotes inefficient polyadenylation [198]. Therefore, TENT4A/B read HBV mRNA as aberrant RNA and further polyadenylate it, allowing selective HBV mRNA processing [199]. It holds true that viral mRNAs containing this UAUAAA tail are usually transcribed from an active cccDNA. Unlike the UAUAAA signal located close to the 5′ region in the dslDNA, a non-canonical signal CAUAAA located downstream of the HBs reading frame facilitates the polyadenylation of integrant-derived mRNAs [200]. Although both mRNAs from cccDNA and integrants are translated into identical HBsAg, transcripts from these two distinct templates may have minor difference at their 3′ ends. Therefore, using RNA precipitation and sequencing techniques should be possible to determine what types of viral mRNA TENT4A/B bind exactly and dissect the modes of RG7834 and other inhibitors.

### 8.4. Can RNAi Strategies Achieve Sustained HBsAg Clearance?

There are many advantages to targeting of HBV viral transcripts. First of all, all the transcripts derived from both cccDNA or integrants share a common 3′ fragment (Figure 1). Hence, it is practical to design one oligonucleotide that eliminates the translation of proteins from both templates, which has broader efficacy than single protein-targeted inhibitors. Secondly, HBV has a lower mutation rate than either HCV or HIV. Thus, a pool of two and three oligonucleotides targeting highly conserved regions functions as pan-genotypic therapeutic (Table 2). Thirdly, employing GalNAc conjugation and LNA modification, second-generation small RNAs and SSOs show some hepatocyte-specific delivery and minimal off-target effects in other tissues, where HBV transcription does not occur.

RNAi treatment induced a rapid decline in HBsAg expression, which is an indicator of viral transcription, at least according to interim reports. When the current batch of phase II trials are completed, whether these strategies achieve sustained HBsAg clearance will be determined. Furthermore, whether a rebound of viral transcription takes place will also be determined after the follow-up period. Patients with HBsAg reduced beyond a certain threshold upon RNAi treatment may develop efficient HBsAg-specific T-cell responses and have a higher chance of HBsAg seroconversion. Upon nucleos(t)ide analogue treatment, if HBsAg levels are reduced to <200 IU/mL at the end of treatment, the patient has lower chances of HBV relapse after cessation of treatment, when HBV DNA has been suppressed [201].

## 9. Conclusions

Current perspectives on HBV RNA synthesis suggest that viral transcription could represent a potential antiviral target [80,202], but a systemic review containing all the concepts of existing and ongoing therapeutics in trial that inhibit transcription and impact on the stability of HBV transcripts was not previously available. In this review, we introduce cutting edge knowledge about both cccDNA and integrant templates responsible for viral transcription, list viral and host factors involved in transcription, and summarize all the druggable candidates into two subsets: the ones targeting the synthesis of viral transcripts and the ones decomposing existing transcripts.

To date, NR agonists have shown potent effects in in vitro infection models. Although the RAR agonist Accutane showed no effects on HBV DNA and HBsAg levels in PXB humanized mice, future experiments should focus on other alternative models with more physiological metabolic profiles, comparable to that observed in primary human hepatocytes. Particular attention should be paid to the results of FXR agonist EYP001, when the phase II trial is completed. A number of GalNAc-conjugated siRNA and LNA-SSOs are also being characterized in phase I/II trials. Whether they give rise to rapid and sustained declines in HBsAg levels in patients is currently under investigation.

In summary, although therapeutic targeting of both HBV cccDNA and genomic integrants is challenging, their transcripts share common features, which represent a conserved point of attack. New strategies aimed at inhibition on HBV RNA synthesis could eventually lead to a reduction and some even loss of HBsAg that is difficult to achieve under current therapies for CHB patients. These treatments given alone or as part of combination therapies with nucleos(t)ide analogues may play a role toward a “functional cure” for CHB.

## Figures and Tables

**Table 2:** Inhibitors targeting and silencing HBV transcripts.

Substance	Property	Target	Clinical Status	Effective Dose	Inventor	Reference
RG7834(Ro7020322)	Small molecule	TENT4A/B	Phase I	2–6 nM	Roche	[153,154]
DHQ-1	Small molecule	Unclear	Preclinical	100 nM	Blumberg Institute	[156]
GS-9620(Vesatolimod)	Small molecule	TLR7	Phase II	- ^3^	Gilead	[157,158,159]
GS-9688(Selgantolimod)	Small molecule	TLR8	Phase II	- ^3^	Gilead	[160,161,162]
RG7854	Small molecule	TLR7/8	Phase I	n/a	Roche	Undisclosed
SB 9200(Iranigivir)	Small molecule	RIG-I and NOD2	Phase II	30 mg/kg ^4^	Spring Bank Pharma	[163]
AB-452	Undisclosed	HBV RNA	Phase I ^1^	n/a	Arbutus Biopharm.	[164]
HBVU6no.2	shRNA	HBV S region	Preclinical	<5 μg ^5^	n/a	[165]
HBV765	shRNA	HBV S region	Preclinical	2 × 10^9^ PFU ^6^	n/a	[166,167]
ALN-HBV(ALN-HBV01)	GalNAc-siRNA	n/a	Phase I ^2^	n/a	Alnylam Pharma	[168]
ARC-520 ^§^	GalNAc-Chol-siRNA	HBV X region	Phase II ^2^	<1 mg/kg ^7^	Arrowhead Pharma	[169,170]
ARB-1467(TKM HBV)	siRNA	S and X regions	Phase II	0.4 mg/kg ^8^	Arbutus Biopharma	[171]
VIR-2218(ALN-HBV02)	GalNAc-siRNA	HBV X region	Phase I/II	n/a	Alnylam and VIR	EASL 2020
JNJ-3989(ARO-HBV)	siRNA pool	S and X regions	Phase II	<400mg/month	Arrowhead and Janssen	[172]
AB-729	GalNAc-siRNA	HBV RNA	Phase I	6 nM	Arbutus Biopharma	[173]
RG6346(DCR-HBVS)	GalXc-siRNA	n/a	Phase I/II	n/a	Dicerna Pharma and Roche	AASLD 2020
GSK3228836 (IONIS-HBVRx)	Antisense oligonucleotide	HBV X region	Phase II	n/a	IONIS Pharma and GSK	EASL 2020
GSK3389404 (IONIS-HBVLRx)	Antisense oligonucleotide	HBV X region	Phase II	n/a	IONIS Pharma and GSK	[174]
LNA-Alexa	Oligonucleotide	pgRNA	Preclinical	5 nM	n/a	[175]
Ro7062931(LNA-SSO)	GalNAc-Oligonucleotide	HBV X region	Phase I	≈1 μM	Roche	[168]
Lunar-HBV	UNA oligomer	S and X regions	Preclinical	n/a	Arcturus and Janssen	AASLD 2016
miR-122 mimic	microRNA	HBV RNA	Preclinical	40 nM	n/a	[176,177]

^1^ The clinical trial was discontinued, ^2^ This trial was terminated, ^3^ It shows no direct antiviral activity, but cytokines in conditioned media secreted from human peripheral blood mononuclear cells (PBMCs) treated with GS-9620 at 10 nM have antiviral activity, ^4^ Determined in infected woodchuck model, ^5^ Determined in shRNA-expressing plasmid injected immunocompetent and immunocompromised mice, ^6^ Determined in HBV 1.3.32 transgenic mice transduced with 2 × 10^9^ plaque-forming units (PFU) of adenovirus expressing shRNA (HBV765), ^7^ Determined in immunocompromised NOD-SCID mice, ^8^ Given bi-weekly. n/a: not applicable. ^§^ Consisting of Chol-siHBV-74 and Chol-siHBV-77. Chol: cholesterol-conjugated; GalNAc: N-acetylgalactosamine; LNA: locked nucleic acid; shRNA: small hairpin RNA; siRNA: small interfering RNA; SSO: single-stranded oligonucleotide; UNA: unlocked nucleomonomer agent. TLR: Toll-like receptor; RIG-I: retinoic acid-inducible gene I; NOD2: nucleotide-binding oligomerization domain-containing protein 2. GSK: GlaxoSmithKline. EASL: European Association for the Study of the Liver. AASLD: American Association for the Study of Liver Diseases.
